# Understanding the Representative Gut Microbiota Dysbiosis in Metformin-Treated Type 2 Diabetes Patients Using Genome-Scale Metabolic Modeling

**DOI:** 10.3389/fphys.2018.00775

**Published:** 2018-06-25

**Authors:** Dorines Rosario, Rui Benfeitas, Gholamreza Bidkhori, Cheng Zhang, Mathias Uhlen, Saeed Shoaie, Adil Mardinoglu

**Affiliations:** ^1^Science for Life Laboratory, Royal Institute of Technology, Stockholm, Sweden; ^2^Centre for Host-Microbiome Interactions, Dental Institute, King’s College London, London, United Kingdom; ^3^Centre for Translational Microbiome Research, Department of Microbiology, Tumor and Cell Biology, Karolinska Institute, Stockholm, Sweden; ^4^Department of Biology and Biological Engineering, Chalmers University of Technology, Gothenburg, Sweden

**Keywords:** gut microbiota, dysbiosis, host–microbiome interactions, genome-scale metabolic models, systems biology

## Abstract

Dysbiosis in the gut microbiome composition may be promoted by therapeutic drugs such as metformin, the world’s most prescribed antidiabetic drug. Under metformin treatment, disturbances of the intestinal microbes lead to increased abundance of *Escherichia* spp., *Akkermansia muciniphila, Subdoligranulum variabile* and decreased abundance of *Intestinibacter bartlettii*. This alteration may potentially lead to adverse effects on the host metabolism, with the depletion of butyrate producer genus. However, an increased production of butyrate and propionate was verified in metformin-treated Type 2 diabetes (T2D) patients. The mechanisms underlying these nutritional alterations and their relation with gut microbiota dysbiosis remain unclear. Here, we used Genome-scale Metabolic Models of the representative gut bacteria *Escherichia* spp., *I. bartlettii, A. muciniphila*, and *S. variabile* to elucidate their bacterial metabolism and its effect on intestinal nutrient pool, including macronutrients (e.g., amino acids and short chain fatty acids), minerals and chemical elements (e.g., iron and oxygen). We applied flux balance analysis (FBA) coupled with synthetic lethality analysis interactions to identify combinations of reactions and extracellular nutrients whose absence prevents growth. Our analyses suggest that *Escherichia* sp. is the bacteria least vulnerable to nutrient availability. We have also examined bacterial contribution to extracellular nutrients including short chain fatty acids, amino acids, and gasses. For instance, *Escherichia* sp. and *S. variabile* may contribute to the production of important short chain fatty acids (e.g., acetate and butyrate, respectively) involved in the host physiology under aerobic and anaerobic conditions. We have also identified pathway susceptibility to nutrient availability and reaction changes among the four bacteria using both FBA and flux variability analysis. For instance, lipopolysaccharide synthesis, nucleotide sugar metabolism, and amino acid metabolism are pathways susceptible to changes in *Escherichia* sp. and *A. muciniphila*. Our observations highlight important commensal and competing behavior, and their association with cellular metabolism for prevalent gut microbes. The results of our analysis have potential important implications for development of new therapeutic approaches in T2D patients through the development of prebiotics, probiotics, or postbiotics.

## Introduction

Dysbiosis in the gut bacterial community and concomitant metabolic changes have an impact on human health (Qin et al., 2012; Tremaroli and Bäckhed, 2012; Karlsson et al., 2013; Forslund et al., 2015; Mardinoglu et al., 2016; Magnusdottir et al., 2017). Gut microbiome could affect host metabolism (Brillat-Savarin, 1826; Tremaroli and Bäckhed, 2012; Shoaie et al., 2013, 2015; Magnusdottir et al., 2017) through degrading non-enzymatically digestible foods, and synthesis of amino acids and short chain fatty acids (SCFAs). Dysbiosis may have detrimental effects on host metabolism such as alterations in abundance of nutrients crucial for homeostasis including butyrate (Forslund et al., 2015; Mardinoglu et al., 2016; Wu et al., 2017). Perturbations of intestinal microbiota are recognized as a risk factor for type 2 diabetes (T2D), a complex chronic disorder associated with genetic and environmental risk factors such as age, diet, and lifestyle (Karlsson et al., 2013; Forslund et al., 2015; Shoaie et al., 2015; Mardinoglu et al., 2016; Magnusdottir et al., 2017). Recently, compositional shifts in representative gut microbes were identified in T2D patients undergoing metformin treatment, the most prescribed antidiabetic drug. These patients display increased abundance of *Escherichia* sp., *Akkermansia muciniphila* (*A. muciniphila*), and *Subdoligranulum variabile* (*S. variabile*) (Forslund et al., 2015; Mardinoglu et al., 2016; Wu et al., 2017), and lower of *Intestinibacter bartlettii* (Forslund et al., 2015; Wu et al., 2017), as well as increased levels of the SCFAs butyrate and propionate. Thus, despite potentially detrimental effects of gut microbiota dysbiosis, metformin-treated patients display beneficial alterations in gut SCFA abundances (Forslund et al., 2015; Mardinoglu et al., 2016). However, the relationship between the metabolism of representative gut bacteria such as *Escherichia* sp., *A. muciniphila, S. variabile* and *I. bartlettii*, and compounds in the intestinal lumen such as SCFAs or amino acids is unclear.

Clarifying complex metabolic responses and relationships between gut microbes and host metabolism requires an analysis of large and highly intertwined reaction networks. GEnome-scale Metabolic models (GEMs) allow for the analysis of such complex networks and have successfully been applied to clarify the mechanisms underlying insulin resistance (Varemo et al., 2015; Zhang and Hua, 2016; Mardinoglu et al., 2018; Turanli et al., 2018) and to identify important nutritional interactions between gut microbes and the host (Shoaie et al., 2013; Ji and Nielsen, 2015; Mardinoglu et al., 2015; Zhang and Hua, 2016). Synthetic lethality analysis (Pratapa et al., 2015) is an approach commonly used in constraint-based modeling to clarify biological phenomena (Mardinoglu and Nielsen, 2012; Mardinoglu et al., 2016; Magnusdottir et al., 2017). It is used to identify vital interconnected metabolic processes underlying a phenotype of interest (Qin et al., 2012; Shoaie et al., 2013; Magnusdottir et al., 2017) and has been extensively applied in health and disease (O’Neil et al., 2017). While synthetic lethality analysis traditionally seeks to identify genes that are individually essential, this approach may assist in identifying whether the simultaneous knock-out of two genes of interest leads to cell lethality, but their individual knock-out maintains cell viability, i.e., synthetic lethality interactions (Kaelin, 2005).

Through reconstruction and analysis of GEMs, we sought to understand the contribution of the four bacteria in the physiology of T2D patients undergoing metformin treatment. We used AGORA GEM reconstructions of *Escherichia* sp., *A. muciniphila, S. variabile*, and *I. bartlettii* to analyze relationships between the bacterial metabolism and the extracellular environment, as well as predicting the survivability of the bacteria against nutritional alterations (Magnusdottir et al., 2017). Here, we employed the concept of synthetic lethality analysis to identify sets of individual and pairs of reactions that, when not present, abolished growth. Additionally, we implemented nutritional interactions analysis to understanding how the presence or absence of gut nutrients influences bacterial growth by focusing on nutrient transport reactions (i.e., exchange reactions). Moreover, we assessed the influence of available nutrients and synthetic lethal reactions on cellular metabolic pathways to clarify which metabolic pathways were mostly dependent on nutritional alterations and under survivability threat, respectively. Lastly, interactions between the gut microbiota and the environment (host intestine) were evaluated through a novel approach based on the production and consumption of substrates of interest under maximal growth and minimal media conditions of each organism. Our observations highlight important association between cellular metabolism of these four prevalent gut microbes and point important implications for development of new therapeutic approaches in T2D patients.

## Materials and Methods

### Genome Scale Metabolic Model Retrieval, Curation, and Modeling

AGORA (Assembly of Gut Organisms through Reconstruction and Analysis) model reconstructions (Magnusdottir et al., 2017) were downloaded in SBML format from Virtual Metabolic Human (VMH) database^1^ for *Escherichia* sp. 4_1_40B, and *I. bartlettii* (*Clostridium bartlettii* DSM 16795) on the 27^th^ of January 2017, for *A. muciniphila* ATCC BAA-835 and *S. variabile* DSM 15176 on the 2^nd^ April 2018. Details regarding microorganism AGORA reconstructions are accessible in **Supplementary Table S1**. The models were manually curated to ensure biological functionality. The computations were performed on resources provided by the Swedish National Infrastructure for Computing (SNIC) through Uppsala Multidisciplinary Center for Advanced Computational Science (UPPMAX).

The RAVEN (Reconstruction, Analysis and Visualization of Metabolic Networks) Toolbox (Agren et al., 2013) was used to define and set parameters for simulations and perform analyses of the originated predictions. Unless otherwise stated, all flux balance analyses (FBAs) considered biomass production as objective function. For flux variability analysis (FVA), minimum and maximum flux ranges were calculated for each reaction for the optimized value of the objective function through the COBRA (Constraint-Based Reconstruction and Analysis) Toolbox (Schellenberger et al., 2012).

### Synthetic Lethality Analysis

Lethality analysis was performed by adapting the Fast-SL algorithm (Pratapa et al., 2015) from the COBRA Toolbox (Schellenberger et al., 2012) to RAVEN Toolbox (Agren et al., 2013). Fast-SL-derived single and double lethal reactions predictions (**Supplementary Table S2**) were further validated by constraining methods, setting lower and upper bounds to zero, with biomass maximization defined as objective function. Single lethal reactions were determined and treated as essential reactions for cell growth. Double lethal reactions were considered as those pairs of reactions that induce no growth when blocked simultaneously but not individually. Exchange reactions were determined using default RAVEN functions, and only those involving nutrient exchange with the extracellular space are reported (i.e., *outside reactions*, and not *inside reactions* which include DNA replication, RNA transcription, protein biosynthesis and biomass, and are treated as intracellular reactions). This permits the identification of essential exchange reactions, which are the nutrients required to be uptaken from the environment by the organism in order to guarantee cell survival.

### Metabolic Pathway Sensitivity to Essential Reactions and Nutrient Changes

The built-in subsystems of the model were used for defining the pathways (**Supplementary Table S5**) and unclassified pathways were ignored. We applied modeling-constraints (lower and upper bounds set to zero and objective function defined as biomass maximization) going through each of the single and double lethal reactions (essential reactions) and non-essential exchange reactions. Pathway sensitivity to changes was determined based on the proportion of reactions that presented absolute flux changes above 0.01 mmol/gDW/h relative to the respective flux in the reference model where no constraints were set on lower/upper bounds. This value was conservatively considered based on the observation that FBA-based approaches often use 0.001 mmol/gDW/h as threshold for identifying reactions that have fluxes (Hyotylainen et al., 2016).

Additionally, these results were compared with those from FVA in response to the inhibition of single and paired synthetic lethal (essential) and non-essential exchange reactions, and compared to a reference output without applied constraints on lethal neither exchange reactions. Only solutions on flux variation that achieve ≥90% of the reference solution were considered. Using the minimum and maximum fluxes determined for each reaction, we computed the mean and ranges for all reactions in each subsystem.

### Extracellular Nutrient Uptake and Alternative Aerobic and Anaerobic *Escherichia* sp. Growth

We have employed a novel approach which allowed us to identify which are the minimal sufficient nutrients that when combined are capable of providing cellular growth when uptaken by the organism. In order to identify which nutrients are on the first line promoting cellular growth under environmental limited conditions, the target reactions of this approach were non-essential exchange reactions. No constraint was applied for single essential exchange reactions to ensure that growth inhibition was not due to the block of required essential nutrients. Cellular intake through non-essential exchange reactions was blocked with lower bounds set to zero. Based on FBA methods, non-essential exchange reactions were blocked one-by-one, two-by-two, and three-by-three. Future work should test how this approach compares with existing methods for determining minimum growth conditions (e.g., Imielinski et al., 2006; Eker et al., 2013). This was performed for all organisms under anaerobiosis, and also for *Escherichia* sp. under aerobiosis. A biomass flux threshold of 10^-5^ was defined as minimum to consider cell growth.

### Maximal Growth-Coupled Extracellular Nutrient Production and Consumption

We developed a novel approach to assess the contribution of each bacteria for nutrient production and consumption under the maximum growth rate permitted under minimum media conditions. Specifically, we determined the maximal rate of secretion or intake of each metabolite when the organism is at its highest growth yield by individually setting each metabolite of interest as objective function at a time, therefore maximizing its production or consumption. Maximum organism growth was determined based on FBA under minimal media for each organism (**Supplementary Table S7**). Thus, the predicted maximal growth (0.6387, 0.2268, 0.2599, 0.2460 mmol/gDW/h, respectively for *Escherichia* sp., *I. bartlettii, A. muciniphila*, and *S. variabile*) was used as lower bound constraint for biomass together with minimal media conditions and under anaerobic conditions (with oxygen exchange constrained to zero in both models).

## Results

### *In Silico* Identification of Different Growth Requirements in Representative Gut Bacteria

**FIGURE 1:**
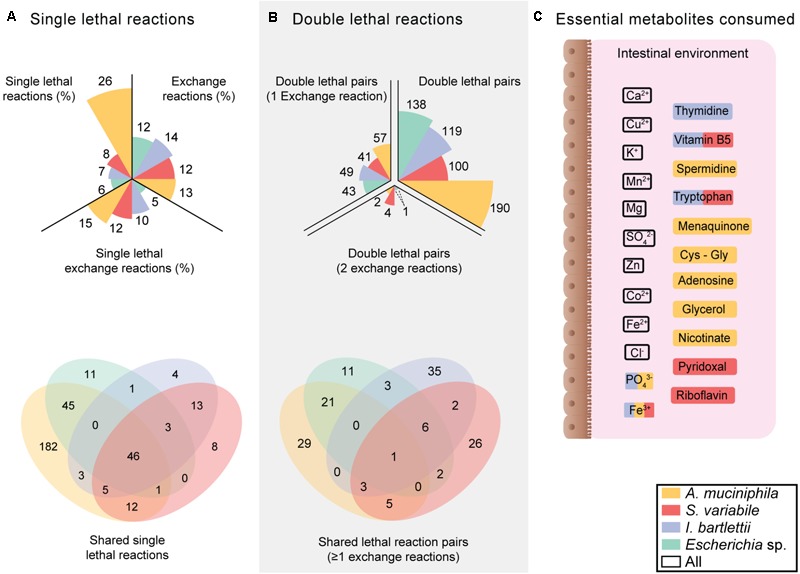
Specific growth requirements suggest lower vulnerability of *Escherichia* sp. to environmental nutritional deficiencies. **(A)** Proportions of single lethal reaction sets for the entire metabolic network and for exchange reactions in the four organisms, and number of exclusive and shared single lethal reactions (**Supplementary Table S2**) **(B)** Number of double lethal reaction pairs. **(C)** Essential metabolites consumed by the four organisms. Multiple colored metabolites are consumed by several bacteria according to the legend.

To assess growth requirements of *Escherichia* sp., *I. bartlettii, A. muciniphila* and *S. variabile*, we retrieved AGORA (Magnusdottir et al., 2017) models for these organisms (**Supplementary Table S1**). These models comprise the entire known metabolic reaction networks of these organisms, and contain 1757, 1095, 1125, and 1057 reactions, and 1267, 730, 592, and 1313 genes, respectively. Using the FAST-SL algorithm (Pratapa et al., 2015) based on FBAs) with biomass as objective function, we performed synthetic lethality interaction analysis (**Figure 1**) on these four organisms. Through this approach, we revealed the influence of an inhibited (i.e., without flux) reaction on the metabolic network. This allowed for the identification of single essential reactions (**Figure 1A**), and those combinations of reaction pairs that become lethal when blocked simultaneously but not individually (**Figure 1B**). In total, this represents between 559,153 to 1,544,403 different conditions (including single and double reaction combinations) tested.

Additionally, this approach allowed for understanding the consequences of unavailability of environmental compounds (e.g., amino acids or oxygen) on cell growth by inhibiting transport reactions with the extracellular environment (i.e., exchange reactions). *Escherichia* sp., *I. bartlettii, A. muciniphila*, and *S. variabile* respectively displayed 211, 153, 142, and 130 exchange reactions. *I. bartlettii* was the bacteria with higher proportion of exchange reactions, while *S. variabile* was the organism with higher proportion of single lethal reactions. *Escherichia* sp. was the bacteria with lower proportion of essential exchange reactions (**Figure 1A** and **Supplementary Table S2**). These four organisms commonly shared 46 single lethal reactions. *A. muciniphila* presented 182 organism specific single lethal reactions and 45 additional single lethal reactions shared with *Escherichia* sp. Among all exchange reactions, 10 single-lethal were shared by these four organisms: environmental exchange of calcium, chloride, carbon dioxide, copper, potassium, magnesium, manganese, sulfate, zinc, and ferrous (Fe^2+^) iron (**Figure 1C**). *Escherichia* sp. did not present organism-specific essential exchange reactions, whereas *I. bartlettii, S. variabile*, and *A. muciniphila* respectively had 1, 2, and 6 single-lethal exchange reactions found only in these organisms. Both *A. muciniphila* and *S. variabile* presented shared single-lethal exchange reactions with *I. bartlettii*, where exchange of ferric iron (Fe^3+^) was essential in the three organisms. Exchange of vitamin B5 and tryptophan were essential exchange reactions found in *I. bartlettii* and *S. variabile*, whereas exchange of hydrogen phosphate is commonly essential in *I. bartlettii* and *A. muciniphila.*

When considering all possible pairs of combinations, *A. muciniphila* presented the highest proportion of double lethal reaction pairs and pairs that include at least 1 exchange reaction. *I. bartlettii* was the organism with higher number of organism-specific lethal reaction pairs with ≥1 exchange reaction, followed by *A. muciniphila* (**Figure 1B**). There were no lethal reaction pairs (≥1 exchange reactions) exclusively shared by the two organisms. However, ornithine exchange comprised 7 and 4 organism-specific double lethal reactions in *I. bartlettii* and *A. muciniphila*, respectively. Among those combinations found together with ornithine exchange, the urea cycle was the only common pathway between the two bacteria, where arginine and proline metabolism are specific for *A. muciniphila*, and alanine and aspartate metabolism, pyrimidine synthesis, citric acid cycle are specific for *I. bartlettii*.

Intracellular reactions involving NADP^+^/NADPH became lethal when combined with riboflavin or diaminoheptanedioate exchange in *I. bartlettii*. In turn, intracellular reactions involving NADP^+^/NADPH together with environmental exchange of vitamin B_5_, the fatty acid laurate or thymidine were synthetic lethal reaction pairs in *Escherichia* sp., but not in *I. bartlettii. Escherichia* sp. displayed the lowest proportion of double lethal reactions, as well as the lowest proportion of double lethal reactions with ≥1 exchange reactions, and the lowest number of organism-specific lethal reaction pairs with ≥1 exchange reactions. Among these pairs with ≥1 exchange reaction which involved fatty acids, *Escherichia* sp. and *A. muciniphila* respectively displayed 20 of 21 shared reaction pairs involving laurate exchange and an intracellular reaction associated with fatty acid synthesis or oxidation. Simultaneous inhibition of acetate exchange and acetate kinase or phosphotransacetylase reactions were lethal in *A. muciniphila.*

Several double lethal pairs involving nicotinate exchange were present in *S. variabile* (three pairs) and *I. bartlettii* (four pairs). Lethal pairs involving nicotinamide mononucleotide (NMN) exchange were also found for *S. variabile* (three pairs) and *Escherichia* sp. (two pairs), where one pair involves nicotinate-nucleotide adenylyltransferase in both organisms. Reactions involved in hypoxanthine exchange and purine synthesis were found in 10 double lethal pairs exclusive of *S. variabile*.

The inhibition of L-lysine exchange simultaneously with diaminopimelate decarboxylase reaction was the only lethal pair found in the four organisms. Reaction pairs including exchange of arginine, alanine, asparagine, aspartate, isoleucine, lysine, tyrosine, valine, thymidine, or thiamine (vitamin B1) were synthetic lethal in one or more organisms. *S. variabile, A. muciniphila, Escherichia* sp., and *I. bartlettii* respectively had 4, 2, 1 and 1 double lethal pairs involving 2 exchange reactions. Simultaneous inhibition of NMN exchange and nicotinate, or L-tyrosine coupled with glycyl-L-tyrosine, or phosphate paired with glycerol 3-phosphate and uracil paired with succinate became lethal in *S. variabile.* In *A. muciniphila* inhibiting the exchange of L-asparagine together with glycyl-L-asparagine or thiamin led to lethality. Notably, simultaneous blocking of exchange of oxygen with ferric iron (Fe^3+^) or nicotinate, respectively prevented growth in *Escherichia* sp. and *I. bartlettii*. While *I. bartlettii* is an obligate anaerobe (**Supplementary Table S1**), the observation that O_2_ exchange is present in this model could indicate that the model failed to describe its aerotolerance. However, the two following points indicate that the model predictions are robust to O_2_ availability. First, the Spearman correlation between model fluxes in presence vs. absence of O_2_ was very high (Spearman’s ρ > 0.82, *P* < 10^-70^ considering all 304 non-null fluxes of both models). Second, the reactions catalyzed by antioxidants against reactive oxygen species (hydrogen peroxide reductase) showed activity in a model encompassing oxygen exchange, but not in its absence (**Supplementary Table S3**). We finally, removed oxygen exchange from the *I. bartlettii* model and repeated the lethality analysis for the entire reaction network. The comparison of synthetic lethality analysis under aerobic versus anaerobic conditions changes the number of single lethal reactions from 80 to 85, and from 124 to 171 lethal pairs (**Supplementary Table S4**), respectively. However, only one additional single lethal exchange reaction (methionine exchange) was identified in the *I. bartlettii* model. These observations reinforce the confidence in the predictions of the model in terms of environmental dependency or synthetic lethal reactions.

### Identification of Sensitive Pathways to Inhibition of Lethal and Non-essential Exchange Reactions

**FIGURE 2:**
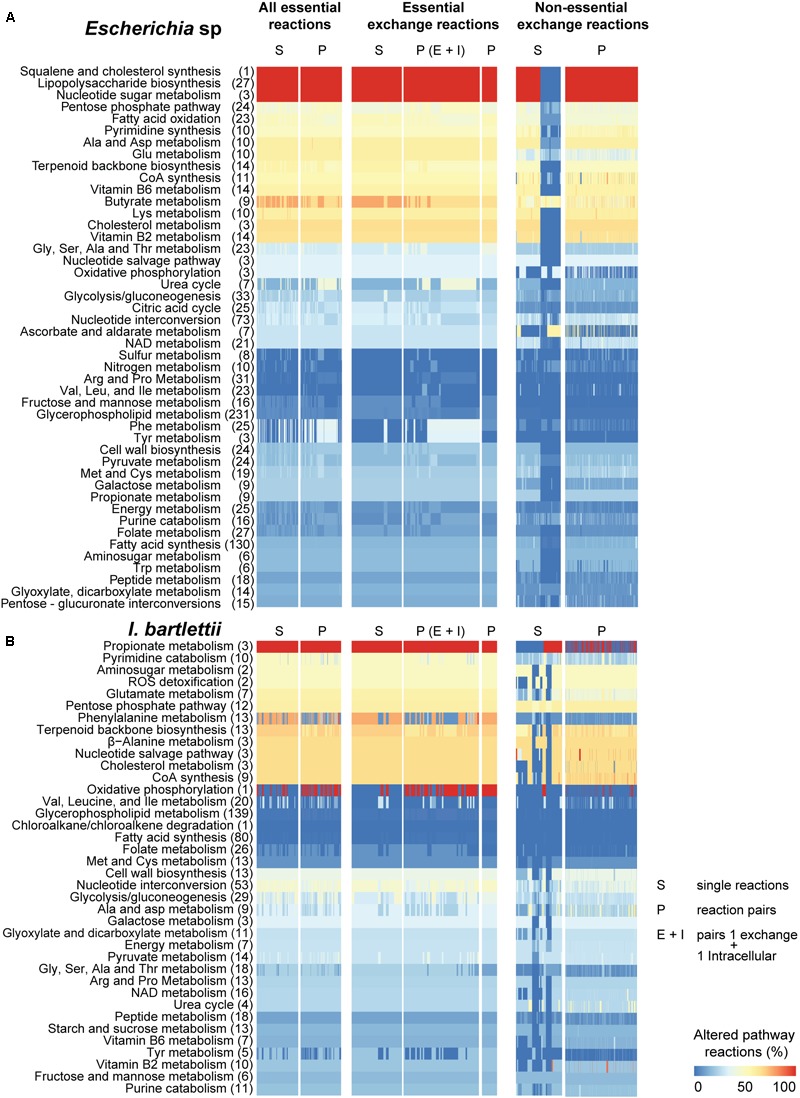
Pathways of *Escherichia* sp. and *Intestinibacter bartlettii* show distinct vulnerability to environmental nutritional changes. Synthetic lethality analysis was performed in *Escherichia* sp. **(A)** and *I. bartlettii* **(B)** for blocking of single reactions or pairs of reactions belonging to the entire metabolic network (“All essential reactions”), for exchange reactions, or for non-essential exchange reactions and then we determined the fraction of pathway reactions altered (>1% change with respect to the reference model). For reaction pairs, we also considered pairs comprising 1 exchange reaction and 1 intracellular reaction. Columns have different number of blocked reactions, and only one pair of essential exchange reactions was found in each organism (see text). Columns leading to no pathway changes are not shown; for non-essential exchange reactions, only those in the top 30% inducing most pathway changes are shown. Amino acids are abbreviated by their common three-letter names. Total number of reactions in each pathway are presented in brackets. Due to the large number of possible combinations, only those pairs of non-essential exchange reactions that resulted in high pathway changes are shown (sum over all pathways >4).

**FIGURE 3:**
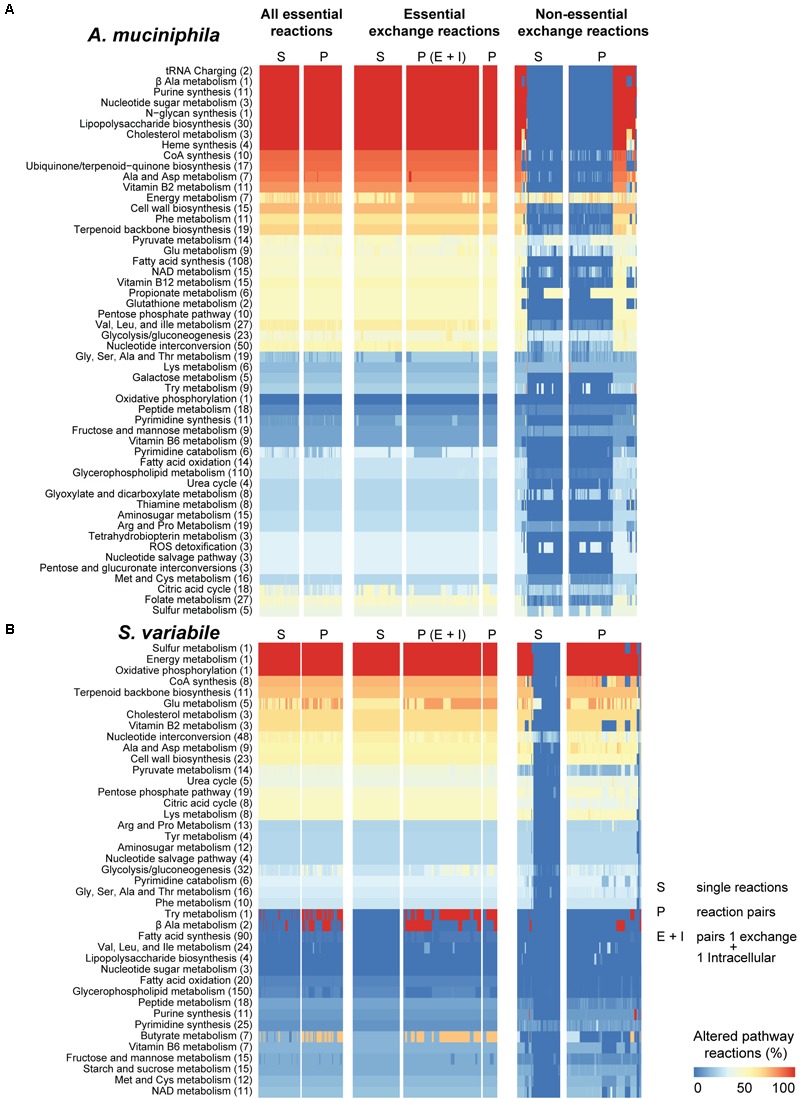
Pathway susceptibility to environmental changes in *Akkermansia muciniphila* and *Subdoligranulum variabile*. Synthetic lethality analysis was performed in *A. muciniphila* **(A)** and *S. variabile* **(B)** similarly to **Figure 2**. Amino acids are abbreviated by their common three-letter names. Total number of reactions in each pathway are presented in brackets. Due to the large number of possible combinations, only those pairs of non-essential exchange reactions that resulted in high pathway changes are shown (sum over all pathways >4).

We investigated which pathways were mostly altered by single and double synthetic lethal reactions (i.e., essential reactions) and non-essential exchange reactions. *Escherichia* sp. displays 73 metabolic pathways, *I. bartlettii* displays 66, *A. muciniphila* has 68 and *S. variabile* displays 63, of which 54 are commonly present in the four organisms (**Supplementary Table S5**). Considering the entire metabolic network and sets of single, paired essential reactions, and non-essential exchange reactions individually and coupled in pairs, we computed the proportion of reactions that are altered in each pathway in comparison with each bacteria’s reference model, i.e., the model with no reaction blocking. To do so, we used FBA to identify flux distribution between pathways while maximizing for bacterial growth, i.e., “pathway sensitivity” to reaction blocking. Additionally and to complement this methodology, we employed FVA (**Supplementary Table S6**). We observed (**Figures 2, 3**) that several pathways show significant alterations in >50% of their reactions. For instance, cholesterol (and squalene) synthesis but not other reactions involved in cholesterol metabolism, were highly perturbed by essential reactions and partly by environmental exchange reactions in *Escherichia* sp. In turn, cholesterol metabolism was highly perturbed by essential and environmental exchange reactions under the same constraints in *A. muciniphila* but not in *I. bartlettii* and *S. variabile*.

*Akkermansia muciniphila* showed the most significant cellular pathway alterations in response to essential reactions including N-glycan synthesis, exclusive to this bacteria (**Figure 3A**). Lipopolysaccharide (LPS) biosynthesis and nucleotide sugar metabolism were metabolic pathways highly perturbed in *A. muciniphila* and *Escherichia* sp., but not in *S. variabile. I. bartlettii* showed (**Figure 2B**) substantial (>50%) alterations in metabolism of propionate, phenylalanine, alanine but no change in chloroalkane and chloroalkene degradation, a species-exclusive metabolic pathway. In turn, metabolism of butyrate and vitamin B2 showed substantial (>50%) alterations in metabolism in *Escherichia* sp. (**Figure 2A**). Oxidative phosphorylation, a metabolic pathway found in the four organisms, was highly perturbed in *S. variabile* when any of its lethal or non-essential exchange reactions were inhibited. The same was observed in *I. bartlettii*, however mainly when double lethal reactions were blocked simultaneously. The metabolism of sulfur and energy were equally highly perturbed in *S. variabile* in response to inhibition of any of its essential or non-essential exchange reactions, while sulfur metabolism was poorly affected in other species and energy metabolism was only considerably perturbed in *A. muciniphila.*

While some of the pronounced changes exhibited by some pathways reflect their small size (e.g., oxidative phosphorylation with ≤3 reactions), other pathways showed substantial changes though they comprise more reactions. This is the case of LPS biosynthesis (27 reactions in *Escherichia* sp. and 30 reactions in *A. muciniphila*), butyrate metabolism (9 reactions in *I. bartlettii* and *Escherichia* sp.), or phenylalanine metabolism (25 and 10 reactions in *Escherichia* sp. and *S. variabile*). Importantly, these trends were also observed when blocking single or pairs of essential exchange reactions, and for many of the non-essential exchange reactions, indicating the strong effect of nutritional availability in these pathways. Metabolic pathways were more sensitive to inhibition of essential (lethal) reactions that are intracellular and environmentally exchanged, comparatively to non-essential exchange reactions in the four organisms. FVA showed qualitatively similar results, though it indicates that more pathways were sensitive to perturbations than FBA.

### Tyrosine, Phenylalanine, and Vitamin B6 Permit *Escherich*ia sp. Growth Under Aerobic but Not Anaerobic Conditions

**FIGURE 4:**
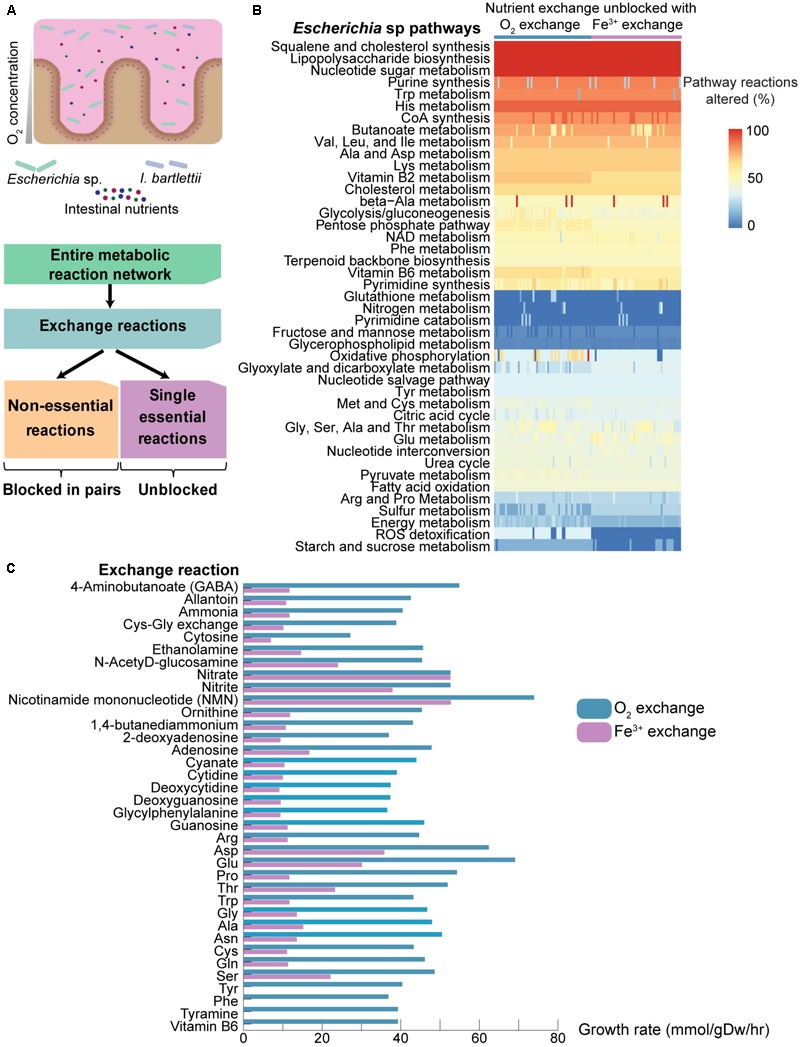
Effect of availability of environmental nutrients on pathway responses and growth rates in *Escherichia* sp. under aerobic and anaerobic growth. **(A)** Due to their aerotolerance, the facultative *Escherichia* sp. may respond differently to environmental nutrients under aerobic versus anaerobic growth, which may provide a selective advantage with respect to the obligate anaerobe *I. bartlettii*. We employed a novel *in silico* approach where all single essential reactions are kept unblocked, and the non-essential exchange reactions (in **Figure 2**) are unblocked one-by-one and two-by-two. This approach may thus assist in identifying those combinations that confer the highest increments on cell growth, as well as determining which pathways most support this response. **(B)** *Escherichia* sp. pathway reactions that are altered (% from total) as response to availability of specific nutrients, together with oxygen or iron exchange (only pairs that either included oxygen or iron exchange resulted in growth). No growth is observed when unblocking single reactions for *Escherichia* sp., or in *I. bartlettii* for single, pairs, or triplets of reactions (results not show). Reactions were considered altered when their flux were altered >1% against the reference model. **(C)** Growth rates for *Escherichia* sp. achieved by unblocking exchange reactions together with O_2_ exchange (i.e., aerobic conditions) or iron exchange (i.e., anaerobic conditions).

Bacteria present different growth requirements, and thus may present selective advantages and disadvantages. Among the four bacteria tested here, all are strict anaerobes with exception to *Escherichia* sp., a facultative aerobe. In *Escherichia* sp., blocking of oxygen and iron exchange together induces lethality (but not individually, since production of ferric iron depends on oxygen through the reaction 4H^+^+ O_2_ + 4Fe^2+^ → 2H_2_O + 4Fe^3+^). We questioned if pathway utilization may differ not only in response to nutrients but also in response to oxygen availability (**Figure 4A**, top). Such differential nutritional responses may present an added selective advantage over anaerobic bacteria.

We investigated pathway response to oxygen availability in *Escherichia* sp., and determined the minimum growth requirements for the four organisms. We developed an approach complementary to those used above for assessing pathway sensitivity (**Figure 4A**, bottom). Briefly, from the entire metabolic reaction network, we selected those involving exchange reactions and blocked all non-essential single exchange reactions identified above, whereas the single-lethal exchange reactions identified above are unblocked. All non-lethal exchange reactions are firstly blocked, and then unblocked one by one, two by two, etc. The synthetic lethality approach employed above optimized for cell growth, and thus allowed for identification of those exchange reactions that most penalize cell growth and whose blocking prevents cell growth using otherwise unconstrained models. In turn, the approach used here optimizes flux distribution in a tightly constrained model and permits identifying those combinations of exchange reactions that, when simultaneously unblocked, promote cell growth. This additionally permits identifying those pathways showing the greatest changes while conferring the greatest increments to cell growth by comparison with the reference fully unconstrained model.

None of the four gut bacteria under study displayed cellular growth when unblocking any single exchange reaction. Only pairs comprising either oxygen or iron exchange resulted in growth for *Escherichia* sp. when combinations of two-by-two non-essential reactions were allowed. In combinations of three exchange reactions, oxygen and iron exchange is always present as one of the necessary reactions for growth (results not shown). Unblocking combinations of two non-essential reactions in *A. muciniphila* and *S. variabile* provided significant cellular growth. Notably, employing this approach yielded no growth in *I. bartlettii* using combinations of 1, 2, and even 3 unblocked non-essential exchange reactions (results not shown), suggesting that more nutrients must be available in order to permit growth. The *Escherichia* sp. model shows growth with as few as 12 exchange reactions (of which 10 are single-essential), whereas the model for *I. bartlettii* does not grow with 18 exchange reactions (including 15 single-essential). Additionally, *A. muciniphila* shows growth with only 23 exchange reactions (which includes 21 single-essential), while *S. variabile* shows growth with only 14 exchange reactions (of which 12 single-essential).

*Escherichia* sp. displays substantial pathway changes (**Figure 4B**) in LPS, squalene and cholesterol biosynthesis, nucleotide sugar metabolism (>90% pathway reactions with >1% fluxes under all assessed conditions), purine and butyrate metabolism (>74% reactions altered), as well as metabolism of histidine, tryptophan, valine, leucine, isoleucine, aspartate, alanine, and lysine (>70%). Glutathione and nitrogen metabolism tend to be mostly unchanged (<2%). Additionally, we observed that *Escherichia* sp. responds differently depending on oxygen availability. As expected from aerobic growth, ROS detoxification is significantly active when O_2_ exchange is unconstrained versus no changes when Fe^3+^ is unblocked but O_2_ exchange is blocked (respectively, >33 vs. 0%, compare **Figure 4B**, left with right). Slight increased fluxes are also identified under aerobic conditions in energy metabolism (mean pathway reaction changes >21% aerobic vs. 16% anaerobic), pentose phosphate pathway (57 vs. 50%), starch and sucrose metabolism (14 vs. 3%), and metabolism of vitamins B6 (64 vs. 57%) and B2 (71 vs. 64%). Oxidative phosphorylation shows substantial increases (>66%) in some entries under aerobic conditions, but not under anaerobic conditions, when exchange of some compounds and amino acids is unblocked (e.g., NMN, alanine, glutamine, glycine, proline, serine, threonine, and tryptophan). In turn, sulfur metabolism (18 vs. 25%) and glyoxylate/dicarboxylate metabolism (29 vs. 35%) are slightly altered under anaerobic conditions. Practically all nutrients confer highest growth rates under aerobic than anaerobic conditions, with exception to nitrate exchange that elicits similar growth rates under aerobic and anaerobic growth. NMN, glutamate, aspartate, nitrate, and nitrite exchange confer the most substantial increases to growth under aerobic and anaerobic conditions (**Figure 4C**). Interestingly, tyrosine, phenylalanine, tyramine, and vitamin B6 uptake allow for cell growth under aerobic but not anaerobic conditions.

### Commensal and Competing Metabolic Behavior of Gut Bacteria in the Utilization of Amino Acids and Short Chain Fatty Acids

**FIGURE 5:**
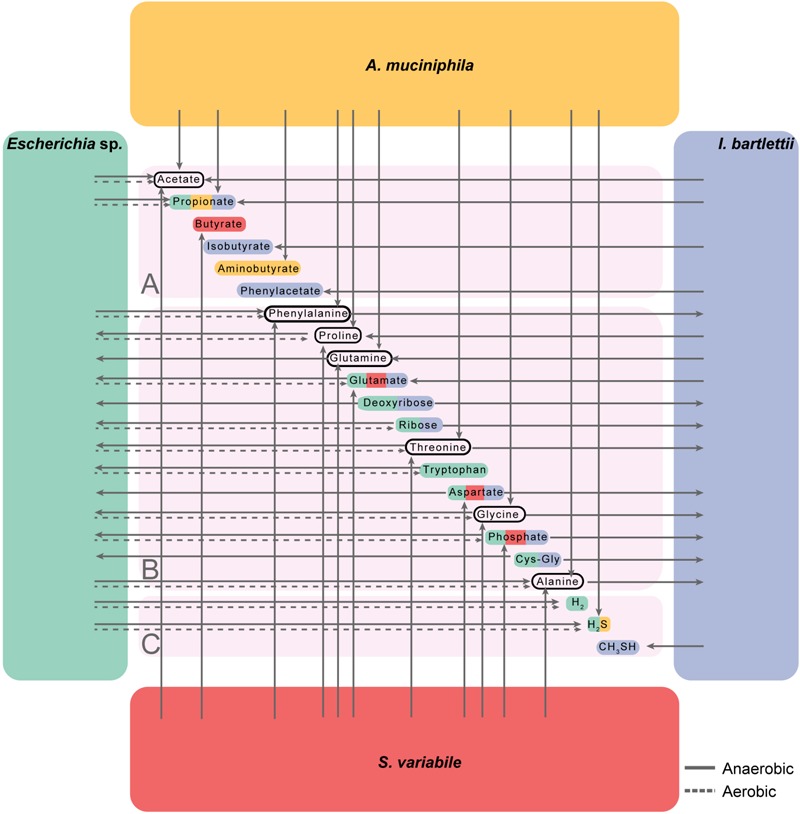
Contribution and competition of the four bacteria for extracellular substrates including short chain fatty acids **(A)**, amino acids and nucleobases **(B)**, and gases **(C)**. Metabolic models for all organisms considered biomass maximization under minimal media anaerobic or aerobic conditions (respectively, solid and dashed arrows, see section “Materials and Methods” and **Supplementary Tables S7, S8**). Cys–Gly indicates Cysteinylglycine. Glutamine, deoxyribose, aspartate, and Cys–Gly show no flux under aerobic growth. Metabolite colors indicate the bacteria that influence its extracellular levels. For all bacteria we considered minimal medium conditions (see section “Materials and Methods”) with exception to *A. muciniphila*, which additionally requires mucin to grow (de la Cuesta-Zuluaga et al., 2017).

We also determined how amino acids, short chain fatty acids, and other nutrients important for host and bacterial metabolism (Shoaie et al., 2013; Forslund et al., 2015; Mardinoglu et al., 2016) were produced or used by the four bacteria. To this extent, we aimed to determine for each metabolite its maximum growth-coupled uptake/secretion fluxes under maximal growth and minimal media conditions in anaerobiosis (**Supplementary Table S7**, see section “Materials and Methods”), since the human intestinal environment is predominantly anaerobic (Tremaroli and Bäckhed, 2012; Donaldson et al., 2015). We observed that the four organisms may contribute for the production of extracellular acetate, whereas all but *S. variabile* produced propionate. Predictions have shown butyrate production by *S. variabile.* In turn, *I. bartlettii* produced isobutyrate (**Figure 5A** and **Supplementary Table S8**), while both *Escherichia* sp. and *I. bartlettii* revealed to compete for ribose, deoxyribose and cysteinylglycine, as well as for aspartate and phosphate, which were both products of *S. variabile* (**Figure 5B**).

Potential commensal behavior may occur, since some of these compounds may be produced by *A. muciniphila* and *S. variabile* (e.g., threonine and glycine), while consumed by *Escherichia* sp. and *I. bartlettii*. Phenylalanine produced by the three other bacteria may be consumed by *I. bartlettii*, which in turn is predicted to secrete phenylacetate. Proline and glutamine were produced by *A. muciniphila, I. bartlettii* and *S. variabile* and consumed by *Escherichia* sp. Finally, *Escherichia* sp. was involved in the production of the gasses hydrogen and, together with *A. muciniphila*, both may produce hydrogen sulfide; whereas *I. bartlettii* produced methanethiol (**Figure 5C**). Because *Escherichia* sp. is a facultative aerobe we repeated these analyses under aerobic conditions, and observed some differences in comparison with the results under anaerobiosis, specifically in the secretion of amino acids (e.g., proline, glutamate, and threonine) and nucleobases (**Figure 5B**).

Altogether, our results demonstrated that the four bacteria displayed substantial differences in substrate requirements for growth, as well as metabolic responses to nutritional changes in the environment. As a consequence of their metabolisms, these four organisms differently contributed and competed for nutrients in the gut, which among those were short chain fatty acids, amino acids, and gasses.

## Discussion

Dysbiosis is one of the main features observed in metformin-treated T2D patients, where there is higher relative abundance of *Escherichia* spp., *A. muciniphila, S. variabile* but lower of *I. bartlettii* (Forslund et al., 2015; Mardinoglu et al., 2016; Wu et al., 2017). Moreover, larger concentrations of the SCFAs propionate and butyrate were reported under drug treatment (Forslund et al., 2015; Mardinoglu et al., 2016; Wu et al., 2017). However, the observation that metformin-treated T2D patients show a depletion in Firmicutes bacteria including *I. bartlettii* (Forslund et al., 2015; Wu et al., 2017), and that Firmicutes and Clostridia are major sources of butyrate (Tremaroli and Bäckhed, 2012; Shoaie et al., 2013, 2015), raises questions about the possible sources of SCFAs. Systems biology approaches have consistently been applied to clarify complex biological processes (Benfeitas et al., 2017; Lee et al., 2017, 2018; Uhlen et al., 2017) including in the relationship between host and gut microbiota (Shoaie et al., 2013, 2015; Forslund et al., 2015; Mardinoglu et al., 2015). Here, we used systems biology methodologies including genome-scale metabolic models and flux balance optimization to clarify the metabolic relationships between the prevalent gut bacteria *Escherichia* sp., *A. muciniphila, S. variabile*, and *I. bartlettii* and their contributions for extracellular pool of compounds including SCFAs and amino acids. Based on synthetic lethality, we also examined the influence of uptake reactions, which involve substrate exchange with the extracellular space, not only on bacterial growth rates but also on flux distribution across intracellular pathways.

The cumulative evidence presented here suggests that the shifts in microbiota diversity reported under metformin treatment and their resulting increase in butyrate and propionate pool (Forslund et al., 2015; Mardinoglu et al., 2016; Wu et al., 2017) may be due to an increased abundance of *S. variabile*, a butyrate-producing anaerobe (Louis and Flint, 2009). *A. muciniphila* may produce aminobutyrate, while *I. bartlettii* produces isobutyrate, a branched chain fatty acid that has been associated with increased risk of colon cancer (Shoaie et al., 2015). In turn, while the enzyme-coding genes involved in butyrate production are present in *Escherichia* sp., this compound is not produced by the wild-type bacterium but may be engineered to do so (Baek et al., 2013). It remains to test if other butyrate-producing bacteria (Forslund et al., 2015) show similar trends. Moreover, our modeling simulations indicate that *I. bartlettii, A. muciniphila*, and *Escherichia* sp. may contribute for the extracellular pool of propionate, of which *A. muciniphila* had previously been observed to produce propionate (Derrien et al., 2004). These observations were associated with the major changing pathways in the four organisms. Propionate metabolism was the pathway displaying the highest responses to alterations in nutrient uptake in *I. bartlettii*, together with metabolism of phenylalanine. In turn, butyrate metabolism in *S. variabile* was perturbed depending on the inhibited reactions.

Bloating and intestinal discomfort are reported side-effects of metformin medication (Forslund et al., 2015), and gasses produced by gut microbiota enhance these adverse side effects (Lewis and Cochrane, 2007; Jahng et al., 2012; Forslund et al., 2015). Colonic transit may be beneficially influenced by the production of hydrogen (Lewis and Cochrane, 2007; Jahng et al., 2012). Our observations show that hydrogen was produced by *Escherichia* sp., which also contributed for hydrogen sulfide production together with *A. muciniphila*. Hydrogen sulfide may have several beneficial effects for both host and gut microbes, displaying anti-inflammatory properties, and promoting smooth muscle relaxation and antioxidant defense (Al Khodor et al., 2017). Future work should experimentally test the contributions of the four bacteria to the extracellular pool of gasses, SCFAs, and amino acids.

The mechanism of action of metformin on glucose metabolism was suggested to be mediated through the bacterial production of SCFAs, where local LPS-triggered inflammation and lower intestinal lipid absorption are side effects of the drug (Forslund et al., 2015; Mardinoglu et al., 2016; Wu et al., 2017). *A. muciniphila* and *Escherichia* sp., two bacteria whose abundance is increased in metformin-treated T2D patients, were the two organisms with the most shared single and double essential reactions as indicated by synthetic lethality. These similar responses among the two bacteria are consistent with the high sensitivity of LPS biosynthesis (a pathway exclusive of both bacteria), and nucleotide sugar metabolism.

Among those nutrients that confer the highest increases to *Escherichia* sp. growth both under anaerobic and aerobic growth are NMN and nitrate exchange, as well as tyrosine, phenylalanine, and tyramine uptake under aerobic conditions. NMN exchange is involved in the coenzyme nicotinamide adenine dinucleotide (NAD) salvage pathway I (Henry et al., 2010; Keseler et al., 2013; Wattam et al., 2017) essential for microbial catabolism and growth (Berríos-Rivera et al., 2002). In turn, nitrate exchange was the only reaction that stimulated similar growth rate under alternative circumstances. Denitrification occurs as part of anaerobic respiration by replacing oxygen as final electron acceptor in the electron transport chain. Nitrate:nitrite antiporters (NarU and NarK) are responsible for the incorporation of nitrate and export of nitrogen (Moreno-Vivian et al., 1999; Keseler et al., 2013). The catabolism of aromatic amino acids is one of the important commensal functions between this bacteria and the host (Díaz et al., 2001; Fuchs et al., 2011), and plays an important role in microbial-mediated food digestion in the intestine (Donaldson et al., 2015; Shoaie et al., 2015). Our observations further suggest that potential commensal behavior may be displayed by *Escherichia* sp. and *I. bartlettii* under anaerobic growth, where on one hand the former produces phenylalanine required by the latter, and on the other hand *I. bartlettii* produces proline, glutamine, and glutamate that are uptaken by *Escherichia* sp. Moreover, phenylalanine, tryptophan, and threonine are essential amino acids which must be ingested by the host for nutritional availability, and which are part of the set minimal sufficient sources promoting cellular growth of *Escherichia* sp. Complementarily, arginine, cysteine, glutamine, glycine, proline, and tyrosine are conditionally non-essential amino acids as well as contributing as first line of sufficient sources for *Escherichia* sp. growth.

The dysbiosis induced by metformin treatment of T2D patients promotes nutritional imbalances (Forslund et al., 2015; Mardinoglu et al., 2016) that may impose fitness disadvantages for specific bacterial taxa. The alterations in relative abundance of *Escherichia* sp. was consistent with our observed growth organism requirements. *Escherichia* sp. is a facultative aerobe and displays a slightly lower number of *essential* uptake reactions and higher number of uptake reactions when compared with the other bacteria under study. Additionally, the former organism is capable of growing while requiring fewer uptake reactions when compared with the other three organisms. Thus, our observations are consistent with *Escherichia* sp. showing a higher robustness to environmental nutrient changes. Together with its aerotolerance and steep oxygen gradient in the gut (Díaz et al., 2001; Bueno et al., 2012; Donaldson et al., 2015), this may confer a selective advantage to *Escherichia* sp. over other gut microbes (Díaz et al., 2001; Magnusdottir et al., 2017), allowing it to grow near the oxygen-rich epithelial surface.

Interestingly, among all exchange reactions, simultaneous blocking of oxygen and ferric iron (Fe^3+^) uptake prevents growth of *Escherichia* sp, whereas ferrous iron (Fe^2+^) uptake is by itself essential. Although iron and other metals may be toxic due to radical formation by reaction with reactive oxygen species [i.e., Fenton reaction (Koppenol, 1993)], it is essential for bacterial growth. Iron is a component of hemic enzymes such as hydroperoxidases and cytochromes, and sensed by the BasS-BasR two-component system involved in LPS modification and anoxic redox control (Bueno et al., 2012). In the absence of oxygen, iron may act as electron acceptor whereby reduction of Fe^3+^ is coupled with oxidation of organic matter (Lovley and Phillips, 1986), and its addition to cell culture promotes growth under anoxia (Bueno et al., 2012). Although one may question whether the observed O_2_/Fe^3+^-associated lethality patterns are plausible considering that Fe^2+^ iron is uptaken by the cell, the oxidation of Fe^2+^ to Fe^3+^ by bacterioferritin requires oxygen (4Fe^2+^ + 4H^+^ + O_2_ → 4Fe^3+^ + 2H_2_O). The essentiality of iron in *Escherichia* sp. has been extensively discussed elsewhere (Braun and Braun, 2002), and is encoded into the biomass equation of *Escherichia* sp. where both redox forms are present.

Overall, our *in silico* observations suggest commensal and competing behavior in the production of extracellular compounds including short chain fatty acids and amino acids, among which the metabolism of *Escherichia* sp., *A. muciniphila, S. variabile*, and *I. bartlettii* may explain the observed features in metformin-treated type 2 diabetes patients. These observations remain to be experimentally tested, though the above observations indicate good agreement with previously known features of these organisms; multiple studies have shown that growth predictions by FBA and gene essentiality prediction are in good agreement with experimental observations (Edwards et al., 2001; Feist et al., 2007). Microbiota modulation approaches based on probiotics, prebiotics, and postbiotics are considered as potential therapies in type 2 diabetes patients. Thus, identification of intestinal bacteria playing a beneficial role or promoting adverse effects on glucose and fatty acids metabolism, will allow the identification of potential microbial targets to improve host metabolism.

## Author Contributions

AM conceived and supervised the study. DR, RB, GB, and CZ designed the experiments. DR performed the experiments. SS assisted in model acquisition and refining of the model. DR and RB analyzed the data and wrote the manuscript. All authors have revised and contributed to the final manuscript.

## Conflict of Interest Statement

The authors declare that the research was conducted in the absence of any commercial or financial relationships that could be construed as a potential conflict of interest.

## Footnotes

1 https://vmh.uni.lu/#microbes/search

## Supplementary Material

TABLE S1 Organism and AGORA reconstruction details.

TABLE S2 Single and double essential reactions in *Escherichia* sp. and *Intestinibacter bartlettii*.

TABLE S3 Flux changes in reactive oxygen species (ROS) reactions of *I. bartlettii* under aerobic and anaerobic conditions.

TABLE S4 Influence of oxygen exchange reaction on Synthetic Lethality Analysis of *I. bartlettii*.

TABLE S5 Organism exclusive and shared cellular metabolic pathways.

TABLE S6 Flux variability analysis for the four bacteria.

TABLE S7 Short chain fatty acids and metabolite consumption (negative flux) and production (positive flux) under maximal growth and minimal media constraints.

TABLE S8 Minimal media constraints applied on *Escherichia* sp. and *I. bartlettii* models.
